# Association of Apolipoprotein B and Adiponectin Receptor 1 Genes with Carcass, Bone Integrity and Performance Traits in a Paternal Broiler Line

**DOI:** 10.1371/journal.pone.0136824

**Published:** 2015-08-31

**Authors:** Valdecy Aparecida Rocha da Cruz, Flávio Schramm Schenkel, Rodrigo Pelicioni Savegnago, Natalia Vinhal Grupioni, Nedenia Bonvino Stafuzza, Mehdi Sargolzaei, Adriana Mércia Guaratini Ibelli, Jane de Oliveira Peixoto, Mônica Corrêa Ledur, Danísio Prado Munari

**Affiliations:** 1 Departamento de Ciências Exatas, Faculdade de Ciências Agrárias e Veterinárias (FCAV),Universidade Estadual Paulista (UNESP), Jaboticabal, São Paulo, Brasil; 2 Department of Animal and Poultry Science, University of Guelph, Guelph, Ontario, Canada; 3 The Semex Alliance, Guelph, Ontario, Canada; 4 Embrapa Suínos e Aves, Concórdia, Santa Catarina, Brasil; University of Bologna, ITALY

## Abstract

Apolipoprotein B (*APOB*) and Adiponectin Receptor 1 (*ADIPOR1*) are related to the regulation of feed intake, fat metabolism and protein deposition and are candidate genes for genomic studies in birds. In this study, associations of two single nucleotide polymorphisms (SNPs) *g*.*102* *A>T* (*APOB*) and *g*.*729* *C>T* (*ADIPOR1*) with carcass, bone integrity and performance traits in broilers were investigated. Genotyping was performed on a paternal line of 1,454 broilers. The SNP detection was carried out by PCR-RFLP technique using the restriction enzymes *HhaI* for the SNP *g*.*729* *C>T* and *MslI* for the SNP *g*.*102* *A>T*. The association analyses of the two SNPs with 85 traits were performed using the restricted maximum likelihood (REML) and Generalized Quasi-Likelihood Score (GQLS) methods. For REML the model included the random additive genetic effect of animal and fixed effects of sex, hatch and SNP genotypes. In the GQLS method, a logistic regression was used to associate the genotypes with phenotypes adjusted for fixed effects of sex and hatch. The SNP *g*.*729* *C>T* in the *ADIPOR1* gene was associated with thickness of the femur and breast skin yield. Thus, the *ADIPOR1* gene seems implicated in the metabolism and/or fat deposition and bone integrity in broilers.

## Introduction

The chicken (*Gallus gallus*) was one of the first domestic animals sequenced and its genome assembly was completed in 2004 [1] by the Washington University Genome Sequence Center and the National Human Genome Research Institute of the United States of America. *Gallus gallus* genome has 38 pairs of autosome chromosomes (5 macrochromosomes, 5 intermediates and 28 microchromosomes) and one pair of sex chromosomes, Z and W [2], with a total of 1.2 Gbp. At 2.5–21 cM/Mb, depending on the chromosome, recombination rates are higher in chickens than in humans or rats, which average 1 cM/Mb and 0.5 cM/Mb respectively [3]. This, plus the great diversity among breeds and strains, has made *Gallus gallus* an animal model for studying the genetic basis of phenotypic traits, capitalizing on the possibility of detecting more segregation than in other species.

Many traits of economic interest are complex and determined by a number of unknown genes. *Loci* that control quantitative traits (QTL) have been associated with a number of traits in chickens, including growth, body composition, egg production, antibody production, disease resistance, and behavior. However, determining causative genes of phenotypic variation is difficult, since each *locus* controls only a fraction of the phenotypic variance of a given trait [3].

Among the molecular markers, single nucleotide polymorphisms (SNPs) are promising for association studies. These markers are abundant and exhibit low mutation rates, which facilitates genotyping [4]. Many studies have been done on the association between SNPs in candidate genes and metabolic pathway in several species [5–10]. Wong et al. [11] identified 2.8 million SNPs in the chicken genome. This abundance of available SNPs may aid in the mapping of causative polymorphisms underlying complex traits in chickens in the future.

Studies on Apolipoprotein B have identified its role in lipid metabolism. This glycoprotein plays an important part in the absorption, assembly and secretion of lipids, including triglycerides and cholesterol. In chickens, Apolipoprotein B is the main component of IDL (intermediate density lipoprotein) and VLDL (very low density lipoproteins) present in plasma cholesterol [12]. In rats and mice, Apolipoprotein B was found in several forms and with diverse functions at different ages by Mcleod et al. [13], who also reported that Apolipoprotein B48, a protein only half the size of Apolipoprotein B100, could mediate both assembly and secretion of chylomicrons (in the intestine) and VLDL (in the liver). In chickens, the Apolipoprotein B (*APOB*) gene may be related to early body growth rates and fat deposition [14].

Another candidate gene associated with fat metabolism in chickens is Adiponectin (*ADIPO*). Adiponectin exerts its action by binding to two specific receptors, *ADIPOR1* and *ADIPOR2*. Both *ADIPOR1* and *ADIPOR2* are seven transmembrane receptors that are structurally and functionally distinct from G-protein-coupled receptors [15]. Adiponectin receptor 1, encoded by the *ADIPOR1* gene, is a major adiponectin receptor that mediates the glucose and lipid metabolism-related effects of adiponectin on target cells [16]. Research based on animal models has shown that *ADIPOR1* overexpression can increse the biological effects of adiponectin [17].

There is evidence that visceral adiposity or age influences the adiponectin plasma levels in chickens. According to Kershaw and Flier [18], the biological effects of adiponectin depend not only on the relative concentrations in circulation and the properties of the different adiponectin subtypes, but also the specific expression of the subtypes on their respective tissue receptors. Maddineni et al. [19] confirmed their presence in skeletal muscle, diencephalon, pituitary gland, liver, ovary and kidney. In addition, *ADIPO* gene may be associated with the initiation and growth processes of adipose tissue deposition in chickens [20, 21].

There are several studies investigating metabolic and genetic mechanisms regulating fat deposition in chickens [22]. Broilers are genetically selected for body weight and muscle growth. However, this genetic selection also leads to an unwanted increase in visceral adiposity [23, 24]. In human studies, fat has been associated to bone disorders [25] and cardiovascular diseases [26] and there is evidence of the interconnection of both these metabolic problems in modern broiler production.

Given the genetic selection process that modern broilers are subject to, it is important to understand both the direct and indirect influence of *APOB* and *ADIPOR1* genes on the development of birds. Therefore, the object of this study was to investigate the association of the SNPs in the *APOB* and *ADIPOR1* genes with carcass, bone integrity and performance traits in broilers. In addition, two different statistical methodologies were used in order to confirm possible associations.

## Material and Methods

### Ethics Statement

This study was performed with the approval of the Embrapa Swine and Poultry Ethical Committee for Animal Use (CEUA) under protocol number 011/201, following international guidelines for animal welfare.

### Population and the collection of data

Phenotypic records were obtained from 1,454 animals from a paternal lineage of broilers. This line has been developed and is owned by the Poultry Genetic Improvement Program from EMBRAPA Swine and Poultry (Brazilian Agricultural Research Corporation, https://www.embrapa.br/en/home). The experimental research centre is located in the city of Concórdia (27° 14' 03" S—52° 01' 40" W), Santa Catarina state, Brazil. This line of broilers has been under development since 1992 and aims to: increase body weight and carcass yield; improve viability, fertility, hatchability, feed conversion, and reduce abdominal fat. The original population was randomly sampled and then 20 males and 100 females were mated to produce an initial population of approximately 1,500 animals.

The birds were housed collectively until 35 days of age and then, in order to evaluate feed conversion, were moved to individual cages from 35 to 41 days. The birds were banded for identification, and fed a three-phase diet; starter from the 1^st^ to 21^st^ day (21% crude protein and 3,150 kcal metabolizable energy), grower from the 22^nd^ to 34^th^ day (20% protein gross and 3,200 kcal metabolizable energy) and finisher from the 35^st^ to 41^st^ day (18.5% crude protein and 3,200 kcal metabolizable energy).

The birds were slaughtered at 42 days of age following a 6 hour fasting. Cervical dislocation was manually applied before bleeding of the neck. Blood samples were taken for DNA extraction during bleeding. Approximately 2 mL of blood was collected in microtubes with 100 mL (10% v/v) of 0.5 M EDTA anticoagulant. The samples were immediately packed in ice and then stored in a freezer at -20°C.

Eighty-five traits related to performance, carcass composition, organs and bone integrity were evaluated (Tables 1 and 2). Yields were expressed as a percentage of the dressed weight relative to the 42 days live weight. An arcsin transformation was applied to the yield traits to normalize theirs distributions. Feed conversion ratio was calculated by dividing feed intake by weight gain in the period from 35 to 41 days of age.

**Table 1:** Carcass traits and corresponding abbreviations used for weight and yields recorded in the broiler line.

Carcass Traits	Abbreviation	
	Weight	Yield
Weight post bleeding and plucking	WPBP	—
Weight of blood and feathers	WBF	YBF
Chilled carcass weight	WCC	YCC
Weight of abdominal fat	WAF	YAF
Weight of head	WHD	YHD
Weight of feet	WFT	YFT
Weight of liver	WLI	YLI
Weight of heart	WHT	YHT
Weight of gizzard	WGZ	YGZ
Weight of wing	WW	YWW
Weight of wing drummettes	WWD	YWWD
Weight of wing middles	WWM	YWM
Weight of wing tips	WWT	YWT
Weight of chilled tibia	WCT	YCT
Weight of thigh	WTH	YTH
Weight of thigh skin	WTHS	YTHS
Weight of thigh meat	WTHM	YTHM
Weight of drumstick	WDS	YDS
Weight of drumstick skin	WDSS	YDSS
Weight of drumstick meat	WDSM	YDSM
Weight of thigh and drumstick meat	WTHDSM	YTHDSM
Weight of breast	WBT	YBT
Weight of breast skin	WBTS	YBTS
Weight of breast meat	WBTM	YBTM
Weight of breast fillet	WBTF	YBTF
Weight of breast bone	WBTB	YBTB
Weight of back	WBAC	YBAC
Weight of neck	WNEC	YNEC
Weight of lungs	WLNG	YLNG
Weight of tibia	W_TIB	YW_TIB
Weight of the femur	W_FEM	YW_FEM

Weight in grams and yield in %.

**Table 2:** Bone integrity and bird performance traits recorded in the broiler line andcorresponding abbreviations used.

Trait	Abbreviation
**Bone integrity traits**	
Tibia length	LG_TIB
Thickness of the tibia	THK_TIB
Femur length	LNG_FEM
Thickness of the femur	THK_FEM
Curvature of the femur	SCORE
Force required to break the femur	FEM_FORCE
Length of the femoral break	FEM_LNG
Area of the femoral break	FEM_AREA
Force required to break the tibia	TIB_FORCE
Length of the tibial break	TIB_LNG
Break area of the tibia	TIB_AREA
Dry matter content of the femur	FEM_DM
Dry matter content of the tibia	TIB_DM
Ash content of the femur	FEM_ASH
Ash content of the tibia	TIB_ASH
**Performance traits**	
Weight at hatching	WHTC
Weight at 21 days of age	W21
Weight at 35 days of age	W35
Weight at 41 days of age	W41
Weight at 42 days of age	W42
Feed intake 35 to 41 days of age	FI35_41
Weight gain 35 to 41 days of age	WG35_41
Feed conversion 35 to 41 days of age	FC35_41

Weight in grams; thickness, distance and length in centimeters; force in kgf/mm^2^.

After bleeding, the animals were scalded in a hot water bath (60°C for 45 s) and the feathers removed mechanically. The carcass weight was calculated by removing the feathers, blood, head, feet, and organs, except the lungs and kidneys. The carcass cuts (breast, drumsticks and thighs with bones and without skin, wings, neck, and back, which corresponds to the dorsal portion of the carcass) were individually weighed. Weight of muscle cuts, separated from skin and bones, and weight of skin with fat were also recorded.

Tibia and femur length were measured between the distal and proximal ends, and thickness in the central region of the bones. Both traits were measured with manual caliper (0.01mm). The bones were kept at about 0°C for 48 hours and then left at room temperature for about an hour to determine flexural strength. The bending test was performed on a TA—XT Plus Texture Analyzer (Texture Technologies Corporation), using the probe TA-92 (Texture Technologies Corporation). The bones were placed in the same position, with the ends resting on two supports spaced 30 mm. The probe travelled 20 mm after touching the sample at 2.00 mm/s test and pretest speed, and 20.00 mm/s post-test speed. The probe touched the sample with programmed weight force of 500 mg. This force was applied to the central area (diaphysis) to determine the flexural strength and rupture modulus. After breaking, the bone fragments were placed in plastic bags, labeled with the sample number and stored at 0°C for 24 hours to determine dry matter and ash.

To determine percent dry matter, the bone fragments were left at room temperature for about one hour. Subsequently, they were placed in pre-weighed porcelain crucible and kept in an oven at 105°C for 16 hours, and then, placed in a desiccator until they reach room temperature and weighed. The dry matter percentage was determined as the ratio of the dry weight to the wet weight of the samples.

Ash was determined immediately after obtaining the dry matter, using the bone fragments. The samples were incinerated in a muffle for six hours. The initial temperature was 350°C for one hour and increased gradually to 450°C and 550°C for one hour and, finally, to 600°C for three hours. Then, the crucibles with the samples were left in the desiccator until reaching room temperature and then weighed. The ash percentage was determined by dividing ash weight by the dry weight of the sample.

### Extraction and quantification of DNA

DNA extraction was performed using the DNAzol reagent (Invitrogen) according to manufacturer recommendations. After extration, the DNA was resuspended in 150 μL ultrapure water, incubated at 37°C for one hour in a water-bath and stored in a freezer at -20°C. A spectrophotometer (Eppendorf BioPhotometer) was used to quantitate the DNA and then, the samples were diluted with ultrapure water to the concentration of 25 ng/μL.

### Gene amplification and genotyping

Specific regions of the *APOB* and *ADIPOR1* genes were amplified with primers for the identification of SNPs. One pair of primers, including regions of exons and introns, was designed for each gene (Table 3). To identify SNPs, gene fragments were sequenced on ABI 3130XL (Applied Biosystems) and subsequently analyzed with Phred/Phrap/Consed/PolyPhred software [27–29]. One SNP per gene was chosen to be genotyped. The SNPs were selected based on the highest sequencing quality and the highest informativeness, i.e. the most polymorphic ones. The PCR protocols for amplication of DNA fragments were standardized as described in Table 3.

**Table 3:** Primer sequence, GenBank accession number, chromosome position, and size of amplicons.

Primers	Primer Sequence	GenBank accession number	Chromosome (position)	Amplicon
*APOB-F*	5’-CTGCCAAAGACTTGCTGTTGGGTT-3’	NC_006090.3	GGA3 (101882031–101916285 bp)	1,099 bp
*APOB-R*	5’-TCTGTGAGGCGTGTAACCAAGTCA-3’			
*ADIPOR1-F*	5’-CCATGCCACACAAATGTGGGTTCT-3’	NC_006113.3	GGA26 (1088478–1094154 bp)	1,023 bp
*ADIPOR1-R*	5’-TGATGTGACTGGAACTGCAGGGA-3’			

To amplify DNA fragment of *APOB* gene, a PCR reaction was performed in 25 μL containing 1X reaction buffer, 2.5 mM of MgCl_2_, 0.4 mM dNTPs, 0.24 μM of each primer, 1.5 U of Taq DNA Polymerase enzyme (Invitrogen, San Diego, CA), and 50 ng of genomic DNA, and finally adjusting the volume to 25 μL by adding ultrapure water. A similar reaction was used for the primer *ADIPOR1*, but 1.6 mM MgCl_2_ was used instead. The PCR reactions were carried out in BigDye Terminator v3.1 Cycle Sequencing Kit (Applied Biosystems, Foster City, CA) using a touchdown PCR protocol under the following condition: denaturing at 94°C for 5 minutes, followed by 35 cycles of denaturing at 94°C for 1 minute, annealing at 59°C for 1 minute and extension at 72°C for 1 minute. The final extension was performed at 72°C for 10 minutes. Electrophoresis using 1% agarose gel was done to visualize the final product.

After PCR, a cleavage reaction was performed with specific restriction enzymes. The NEBcutter program [30] was used to identify the restriction sites and specific enzymes for the selected SNP. Enzyme *MslI* was selected for the evaluation of the SNPs in fragment amplified by the primers for *APOB*, whereas enzyme *HhaI* was selected for analysis of the SNP in the fragment amplified by the primers for *ADIPOR1*.

The cleavage protocol for each enzyme was:

*MslI*: Each reaction used 2 μL of the PCR product with 1 μL of 10X buffer; 1U enzyme *MslI*, and ultrapure water was added to reach 10 μL. The thermal cycler conditions were: one cycle at 65°C for 3.5 hours, followed by one cycle at 80°C for 20 minutes.
*HhaI*: Each reaction used 2 μL of PCR product in 2 μL of 10X buffer; 1U enzyme *HhaI*, and ultrapure water added to a final volume of 10 μL. The reaction occurred in a water bath at a temperature of 37°C overnight.

Finally, the digestion reaction was visualized using a 2% agarose gel stained with 0.01% ethidium bromide. The samples and molecular markers (100 bp DNA Ladder—Promega Corporation) were loaded on the gel and subjected to electric current at 100 V for about a half hour in 1X TBE (Tris-Borate-EDTA). A UV transilluminator was used to compare the sample band pattern to the 100 bp marker bands.

Both phenotypic and genotypic data used in this study are available upon request to Dr. Mônica Côrreia Ledur (Embrapa Swine and Poultry. Address: Rodovia BR-153, Km 110, Distrito de Tamanduá Caixa Postal: 21 CEP: 89700–000, Concórdia, Santa Catarina, Brazil).

### Statistical analyses

Preliminary analyses, including descriptive statistcs, normality test and testing of fixed effects to be included in the models, were done using the *UNIVARIATE* and *GLM* procedures in the Statistical Analysis System (SAS) software [31]. Outliers were identified using Box-plot and were removed from the data set. The descriptive statistics of the studied traits are in Table 4. Genotypic and allelic frequencies were calculated by counting the genotypes and alleles for each SNP and Hardy-Weinberg equilibrium was tested using a chi-square test at 5% significance level.

**Table 4:** Descriptive statistics for the studied traits.

Trait^1^	N	Mean^2^	SD	CV (%)	Minimum	Maximum
WHTC	1448	47.66	3.70	7.77	37.40	61.80
W21	1426	648.43	133.91	20.65	256.00	1034.00
W35	1450	1730.96	202.59	11.70	776.00	2444.00
W41	1443	2219.20	251.91	11.35	1026.00	2922.00
W42	1452	2223.86	260.24	11.70	988.00	2971.00
FI35_41	1443	1091.45	152.48	13.97	508.00	1590.00
WG35_41	1439	488.77	106.57	21.80	128.00	802.00
FC35_41	1439	2.31	0.47	20.16	1.42	5.25
WPBP	1445	2055.89	247.42	12.03	901.00	2764.00
WBF	1441	168.55	26.37	15.65	82.00	293.00
WCC	1436	1639.06	202.05	12.33	661.20	2212.00
WAF	1435	47.36	14.08	29.73	2.50	94.00
WHD	1428	52.55	7.31	13.91	27.90	77.20
WFT	1424	74.66	13.71	18.36	34.30	110.10
WLI	1422	52.34	8.73	16.68	25.40	82.40
WHT	1421	12.40	2.15	17.35	6.30	19.70
WGZ	1423	32.00	6.04	18.86	17.80	56.10
WW	1422	167.40	20.00	11.95	72.00	236.40
WWD	1432	85.71	11.17	13.03	35.80	121.00
WWM	1425	61.91	7.98	12.89	26.20	87.80
WWT	1433	19.78	2.94	14.87	10.00	31.80
WCT	1432	55.65	10.24	18.40	25.40	85.40
WDS	1421	205.87	31.24	15.17	86.20	306.60
WDSS	1427	17.30	4.38	25.31	5.40	36.60
WDSM	1429	132.98	20.67	15.55	52.80	208.00
PFEM	1434	32.51	5.57	17.14	18.00	56.60
WTH	1430	46.23	9.77	21.14	11.60	87.60
WTHS	1427	310.49	46.11	14.85	113.60	464.40
WTHM	1431	231.75	37.78	16.30	81.40	351.20
WBT	1426	500.76	63.48	12.68	211.30	710.80
WBTS	1431	31.38	6.78	21.60	8.40	61.70
WBTM	1431	294.07	42.66	14.51	123.70	428.60
WBTF	1434	77.55	11.85	15.28	35.70	119.10
WBTB	1435	97.91	15.02	15.34	43.50	152.70
WBAC	1425	263.95	35.84	13.58	120.40	370.70
WNEC	1432	119.37	21.52	18.02	35.60	200.40
WLNG	1430	15.31	3.06	19.98	6.60	24.60
WTHDS	1414	516.41	73.98	14.33	200.20	736.60
WDSTHM	1426	364.69	55.91	15.33	137.20	529.40
W_TIB	673	11.75	2.11	17.99	7.14	17.82
W_FEM	672	8.51	1.37	16.08	4.41	12.68
LG_TIB	673	95.29	3.86	4.05	80.00	106.20
THK_TIB	673	8.76	0.92	10.51	6.00	12.90
THK_FEM	673	8.86	0.73	8.26	7.00	11.43
LNG_FEM	672	69.56	3.09	4.45	59.30	78.00
SCORE	673	1.13	0.34	29.94	1.00	2.00
FEM_FORCE	672	28.69	5.78	20.15	13.45	52.68
FEM_LNG	672	2.97	1.15	38.83	1.51	15.74
FEM_AREA	672	42.69	17.33	40.60	11.20	135.74
TIB_FORCE	673	32.06	7.95	24.78	12.86	58.46
TIB_LNG	673	3.62	1.15	31.62	1.90	15.06
TIB_AREA	673	67.96	24.33	35.80	20.21	163.30
FEM_DM	673	51.74	3.53	6.82	43.29	62.86
TIB_DM	672	50.49	3.40	6.73	39.52	64.03
FEM_ASH	673	21.34	1.54	7.20	15.32	28.60
TIB_ASH	673	22.18	1.46	6.58	17.58	28.03

^1^The names of the traits were defined in Tables 1 and 2.

^2^Means of weight (in grams), yields (in percentage), force (in kgf/mm^2^) and thickness (in cm).

N: number of animals; SD: standard deviations; CV: coefficient of variation.

The association of SNPs with the traits was investigated using two different methods: Restricted maximum likelihood via ASREML [32] and Generalized quasi-likelihood scoring (GQLS; [33]).

#### Restricted maximum likelihood method

The ASREML software was used to fit the following univariate linear model for all the traits, which included additive and dominant effects of the SNP markers:

$$y_{ijk}=\mu +sex_{j}+inc_{k}+\overset{2}{\underset{l=1}{\sum }}\beta_{1l}x_{1l}+\overset{2}{\underset{l=1}{\sum }}\beta_{2l}x_{2l}+a_{i}+e_{ijk},$$

where:

*y*_*ijk*_ is observation for the trait on the i^th^ animal,

*μ* is the overall mean,

*sex*_*j*_ is the fixed effect of the j^th^ sex (j = 1, 2),

*inc*_*k*_ is the fixed effect of the k^th^ hatch (k = 1, 2, 3, 4, 5),

*x*_*1l*,_ *x*_*2l*_ are the recoded genotypes (-1, 0, or 1) and (0, 1 or 0) for the l^th^ SNP, i.e. genotypes AA, AT and TT of the *APOB* gene and CC, CT and TT of the *ADIPOR1* gene, respectively,

*β*_1*l*_, *β*_2*l*_ are the linear regression coefficients (additive and dominance effects, respectively) for the l^th^ SNP,

*a*_*i*_ is the random additive genetic (polygenic) effect of the i^th^ animal,

and *e*_*ijk*_ is the residual random effect for the i^th^ animal.

An alternate model was also fit using ASREML to directly estimate the allele substitution effect of the SNPs:

$$y_{ijk}=\mu +sex_{j}+inc_{k}+\overset{2}{\underset{l=1}{\sum }}\beta_{l}x_{l}+a_{i}+e_{ijk},$$

where:

*y*_*ijk*_ is observation for the trait on the i^th^ animal,

*μ* is the overall mean,

*sex*_*j*_ is the fixed effect of the j^th^ sex (j = 1, 2),

*inc*_*k*_ is the fixed effect of the k^th^ hatch (k = 1, 2, 3, 4, 5),

*x*_*l*_ is is the number of copies of a given allele in the genotype of the l^th^ SNP (counted as -1, 0, 1) for genotypes AA, AT and TT of the *APOB* gene and for genotypes CC, CT and TT of the *ADIPOR1* gene, respectively,

β_j_ is the linear regression coefficient (allele substitution effect) for the l^th^ SNP,

*a*_*i*_ is the random additive genetic (polygenic) effect of the i^th^ animal,

and *e*_*ijk*_ is the residual random effect for the i^th^ animal.

All the available pedigree information (1,567 animals) was used to build the numerator relationship matrix for the animal additive genetic effect.

#### Generalized quasi-likelihood score method

The Generalized Quasi-Likelihood Score (GQLS) method developed by Feng et al. [33], which uses a logistic regression model to associate the genotypes (treated as the response variable) with the traits phenotypes adjusted for fixed effects (sex and hatching) was also used an alternative statistical method.

In the GQLS method a logistic regression is used to associate the expected frequency of a marker allele (μ_j_) with the trait phenotypes (X_i_):

$$\mu_{j}=E(Y_{i}|X_{i})=\frac{e^{\beta_{0}+\beta_{1}X_{i}}}{1+e^{\beta_{0}+\beta_{1}X_{i}}},$$

where:

*β*_*0*_ is the intercept and *β*_*1*_ is the linera regression coefficient,

*μ*_*j*_ is expected allele frequence of the j^th^ SNP,

*Y*_*i*_ is proportion of the allele 1 in the observed genotype of the i^th^ animal for j^th^ SNP,

*X*_*i*_ is the phenotypic observation of the i^th^ animal.

The GQLS method accounts for the relatioships among animals through the numerator relationship matrix [33].

The significance of statistical tests needs to be adjusted for multiple hypotheses test, such as in the case of a large number of traits tested in this study. Adjustments can be made by the Bonferroni correction [34]. However, Bonferroni adjustment assumes that all tests are independent. A principal component analysis of the correlation matrix among the 85 traits analyzed in this study was performed using the PRINCOMP procedure in SAS software [31]. The first 31 principle components explained 99% of the total variance. The comparison-wise significance level for Bonferroni adjusment was then α/31, where 31 was considered the effective number of independent tests [35]. An experimental(trait)-wise significant level of 5% was considered to declare a significant association, while a 15% level was considered as a trend for association.

## Results

The two SNPs were found segregating with intermediate allele frequencies and showed high heterozygosity in the broiler line considered (Table 5). The heterozygote frequency was 57.4% (762 animals) for the SNP in the *APOB* gene and 56.5% (763 animals) for the SNP in the *ADIPOR1* gene. Test for Hardy-Weinberg equilibrium showed an excess of heterozygotes (P<0.01), indicating that the population was not in Hardy-Weinberg equilibrium. For the *APOB* SNP, the T allele was more frequent than the A allele, while for the *ADIPOR1* SNP, the C allele was more frequent than the T allele (Table 5).

**Table 5:** Marker, number of animals (N), genotypic and allelic frequencies for the SNPs studied in the broiler line. Allelic and genotypic frequencies, percentages are between parentheses.

Marker	N	Genotype			Allele	
*APOB g*.*102* *A>T* *SNP*		AA	AT	TT	A	T
	1,328	188(14.1)	762(57.4)	378(28.5)	42.9	57.1
*ADIPOR1 g*.*729* *C>T* *SNP*		CC	CT	TT	C	T
	1,351	342(25.3)	763(56.5)	246(18.2)	53.6	46.4

The traits WAF, YWW, YBAC, WNEC, YBTB, and YNEC were not significantly affected by sex (P>0.05) and the traits LG_TIB, LNG_FEM, THK_FEM and TIB_ASH were not significanty affected by hatch (P>0.05). All other traits were significantly affected by sex and hatch (P<0.05).

Tables 6 and 7 shows the results for traits for which at least a trend of association with SNPs in *APOB* and *ADIPOR1* genes was found (P<0.10) at a comparison-wise level. These Tables also present results adjusted for multiple tests. After Bonferroni correction, only the SNP in *ADIPOR1* gene was significantly associated (P<0.05) with two traits (YBTS and THK_FEM) and showed suggestive association (P<0.15) with WBTS and FEM_FORCE using the REML method (Table 6).

**Table 6:** P-values (P) and estimates for additive (a) and dominant (d) effects on broilers traits from a genotypic model and from an allele substitution (α) effect model with corresponding standard errors (se) for SNPs in the *APOB* and *ADIPOR1* genes, using restricted maximum likelihood method (traits are defined in Tables 1 and 2).

Trait	SNP *g*.*102* *A>T* (*APOB* gene)						SNP *g*.*729* *C>T* (*ADIPOR1* gene)					
	Pa	a(se)	Pd	d(se)	Pα	α(se)	Pa	a(se)	Pd	d(se)	Pα	α(se)
FI3541	0.176		**0.014**	20.47(8.25)	**0.055**		0.536		0.448		0.448	
WDS	0.259		**0.071**		0.118		**0.033**	0.43(0.20)	0.883		**0.030**	0.43(0.20)
WBT	0.865		**0.032**	7.25(3.55)	0.775		0.535		0.756		0.486	
WBTS	0.895		0.721		0.817		**0.004** *	0.88(0.04)	0.415		**0.005**	0.84(0.30)
WBTM	0.915		**0.032**	4.96(2.29)	0.569		0.918		0.457		0.896	
THK_FEM	0.308		0.438		0.344		**0.001** **	0.143(0.039)	**0.041**	-0.095(0.046)	**0.001** **	0.1415(0.0388)
FEM_FORCE	0.524		0.376		0.621		**0.003** *	1.072(0.363)	0.569		**0.004** *	1.065(0.363)
TIB_FORCE	0.269		**0.067**	-1.100(0.596)	0.432		**0.016**	1.119(0.460)	0.534		**0.015**	1.133(0.462)
TIB_ASH	0.452		0.105	-0.1968(0.1204)	0.603		0.229		**0.047**	**0.222(0.111)**	0.213	
YBF	0.088	0.0018 (0.0011)	**0.094**	-0.0020(0.0012)	**0.036**	0.0022(0.0011)	0.376		0.436		0.307	
YCC	0.722		**0.006**	0.0031(0.0011)	0.357		0.256		0.247		0.186	
YDSM	**0.090**	-0.0008(0.0005)	0.606		0.059		0.245		**0.041**	0.0010(0.0005)	0.157	
YDS	**0.042**	-0.0012(0.0005)	0.440		**0.022**	-0.0012(0.0005)	0.692		0.146		0.572	
YW_FEM	0.459		0.403		0.372		0.327		**0.028**	0.0009(0.0004)	0.200	
YBTS	0.627		0.715		0.596		**0.001** **	-0.0016(0.0005)	0.191		**0.002** *	-0.0016(0.0005)
YBTM	0.941		**0.028**	0.0022(0.001)	0.591		0.732		0.339		0.859	
YBTF	0.469		**0.033**	0.0013(0.0006)	0.788		0.907		0.924		0.930	

**Significance at 5% experiment-wise level by Bonferroni correction.

*A trend of significance at 15% experiment-wise level by Bonferroni correction.

**Table 7:** P-values for associations of SNPs in the *APOB* and *ADIPOR1* genes with broiler traits, using a Generalized Quasi-Likelihood Score method (traits are defined in Tables 1 and 2).

Trait	SNP *g*.*102* *A>T* (*APOB* gene)	SNP *g*.*729* *C>T* (*ADIPOR1* gene)
THK_FEM	0.679005	0.00014**
FEM_FORCE	0.784031	0.00161**
YBTS	0.73904	0.0023*
WBTS	0.2934	0.0056
TIB_FORCE	0.3811	0.0161
WDSS	0.0867	0.0337
YDSS	0.4914	0.0500
WHT	0.3863	0.0603
YHT	0.1591	0.0617
YAF	0.1706	0.0732
WAF	0.9691	0.0885
FC35_41	0.3437	0.0895
YNEC	0.2455	0.0898
YFT	0.0408	0.8276

**Significance at 5% experiment-wise level by Bonferroni correction.

*A trend of significance at 15% experiment-wise level by Bonferroni correction.

According to the analysis using the GQLS method, no significant associations with the SNP in *APOB* gene were found too. The SNP in the *ADIPOR1* gene remained associated with THAK_FEM, but also was significanty associated with FEM_FORCE (P<0.05), another bone integrity trait (Table 7).

Even though not significantly at an experimetal-wise level, several associations were found at a comparion wise level of 5%, including associations of the SNP in *ADIPOR1* gene with TIB_FORCE, WDSS and YDSS (Tables 6 and 7).

## Discussion

Sex had no significant effect on abdominal fat, although Mignon-Grasteau et al. [36] reported that body weight selection contributes to an increase of sexual dimorphism. Fat depots are greater in females than in males, but this is caused mainly by the larger adipocytes found in females [37]. Differences between sexes for abdominal fat at 28, 35, 42 and 49 days were observed by Dalanezi et al. [38].

There were no reports in literature of sex effect on YWW, YBAC, WNEC, YBTB, and YNEC; and hatch effect on LG_TIB, LNG_FEM, THK_FEM and TIB_ASH traits.

The SNP in the *ADIPOR1* gene showed a significant additive effect on YBTS and THK_FEM, suggesting that this polymorphism may have a direct influence on fat deposition in the breast and bone integrity. This SNP showed an opposite additive effect on breast skin and in thickness of the femur (Table 6). This indicates that skin fat deposition in carcass cuts (breast) and bone quality may be altered in opposite directions if selection is conducted based on this SNP marker, what potentially might be beneficial, i.e. selecting for the favourable allele for bone integrity would lead to decrease in skin fat deposition. The suggestive asociations (P<0.15) of SNP in the *ADIPOR1* gene with FEM_FORCE and WBTS also suggest that this gene may directly influence on fat deposition and bone integrity.

Even though not significantly at an esperimetal-wise level associations of the SNP in the *ADIPOR1* gene with force required to break the tibia (TIB_FORCE), weight and yield of dramstick skin (WDSS and YDSS) were observed, which corroborate with the significant associations found with yield of breast skin (YBTS) and thickness of the femur (THK_FEM) and suggestive associations with weight of breast skin (WBTS) and force required to break the femur (FEM_FORCE).

No significant SNP association with live weights at different ages and with weights of various cuts was found. However, Hendricks et al. [20] found that young chickens (4 weeks old) had a set amount of plasma adiponectin and that age-related changes or fast growth could lead to the decline of circulating adiponectin levels.

Pisto et al. [39] reported that reduction in plasma adiponectin concentration is indicative of an increase in muscle fiber size. Fast-growing broilers have larger muscle fibers [40] and, therefore, have a smaller amount of adiponectin in the blood. This may be the result of muscle glucose uptake and nutrient deposition. Increasing dietary energy increases adiponectin gene expression in abdominal adipose tissue at 32 days of age, but there is no associated increase at 49 days of age [21]. This may explain the absence of an additive effect of SNP *g*.*729* *C>T* on abdominal fat, if at 42 days of age the adiponectin concentration is lower, then it cannot have an effect on the deposition and/or metabolism of abdominal adipose tissue.

Studies in other species have observed relationships between adiponectin, adipose tissue and bone [41]. The exact way adiponectin works is not yet fully understood. However, it is known that other adipokines, such as leptin, which has a positive correlation with adipose deposition and metabolism, inhibits adiponectin production [25]. According to some authors, such as Sinsigalli et al. [42], Barbato et al. [24] and Gonzales et al. [43], the deposition of subcutaneous and abdominal fat and skeletal disorders in broilers are associated with varying concentrations of hormones. Furthermore, genes such as *APOB* and *ADIPOR1* and the neural control mechanisms of satiety and hunger that regulate feed intake also influence those traits.

The lack of association between *ADIPOR1* and abdominal fat deposition may be due to its expression occurs at an early age, as reported in the literature [14, 20, 21] and, in this study, fat deposition was measured at 42 days of age. Another reason for the non association of *ADIPOR1* with abdominal fat may be due to the fact that this gene influences the deposition of different fatty acids present in breast and drumstick fat. According Crespo and Garcia [44], abdominal fat contains more oleic acid, while the breast and thigh fat contains more stearic acid.

While the results of this study provided essential information and found that the SNP in the *ADIPOR1* gene was significantly associated with bone integrity and fat deposition in the breast its role in the chicken’s metabolism needs to be further studied before it can be used for genetic screening. The role of the adiponectin gene on metabolism must be better understood, as the intense selection pressure for increased body weight affects this hormone. Excess of adipose tissue increases the production of many adipokines that influence several bodily functions in birds.

The SNP in the *ADIPOR1* gene is located in an intron region of the gene. According to Ninov et al. [45], when a SNP occurs in the intron region, it may not be involved directly with the associated trait, but may be connected to other polymorphims located in or around the coding and regulatory regions of the gene. This needs to be further investigated.

## Conclusions

The SNP *g*.*729* *C>T* in the *ADIPOR1* gene was found associated with thickness of the femur and breast skin yield. Thus, the *ADIPOR1* gene seems implicated in the metabolism and/or fat deposition and bone integrity in broilers. Further studies are warranted to elucidate if this SNP could be used as molecular genetic markers in broiler selection.

## References

[1] Wallis JW, Aerts J, Groenen MAM, Crooijmans RPMA, Layman D, Graves TA, et al A physical map of the chicken genome. Nature. 2004; 432: 761–764. 15592415 10.1038/nature03030

[2] Crooijmans RPMA, Vrebalov J, Dijkhof RJM, Van Der Poel JJ, Groenen MA. Two-dimensional screening of the wageningen chicken BAC library. Mamm Genome. 2000; 11: 360–363. 10790534 10.1007/s003350010068

[3] Sawai H, Kim HL, Kuno K, Suzuki S, Gotoh H, Takada M, et al The Origin and Genetic Variation of Domestic Chickens with Special Reference to Jungle fowls *Gallus g*. *gallus* and *G*. *varius* . PLOS ONE. 2010; 5: e10639 10.1371/journal.pone.0010639 20502703 PMC2873279

[4] Chen X, Sullivan PF. Single nucleotide polymorphism genotyping: biochemistry, protocol, cost and throughput. Pharmacogenomics J. 2003; 3: 77–96. 12746733 10.1038/sj.tpj.6500167

[5] Jiang YL, Li N, Fan XZ, Xiao LR, Xiang RL, Hu XX, et al Associations of T→ A mutation in the promoter region of myostatin gene with birth weight in yorkshire pigs. Asian Australas J Anim Sci. 2002; 15: 1543–1545.

[6] Li H, Deeb N, Zhou H, Mitchell AD, Ashwell CM, Lamont SJ. Chicken quantitative trait loci for growth and body composition associated with transforming growth factor-beta genes. Poult Sci. 2003; 82: 347–356. 12705392 10.1093/ps/82.3.347

[7] Zhang BZ, Lei MG, Deng CY, Xiong YH, Zuo B, Li FE. Association between PCR-RFLP polymorphism of the fifth intron in lipoprotein lipase gene and productive traits in pig resource family. Asian Australas J Anim Sci. 2005; 18: 458–462.

[8] Chung ER, Kim WT. Association of SNP marker in *IGF-I* and *MYF5* candidate genes with growth traits in Korean cattle. Asian Australas J Anim Sci. 2005; 18: 1061–1065.

[9] Meng HJ, Zhao G, Li ZH, Li H. Single nucleotide polymorphism on 81 peroxisome proliferators- activated receptor genes associated with fatness traits in chicken. Asian Australas J Anim Sci. 2005; 18: 1221–1225.

[10] Xu DQ, Xiong YZ, Liu M, Lan J, Ling XF, Deng CY, et al Association analyses with carcass traits in the porcine KIAA1717 and HUMMLC2B genes. Asian Australas J Anim Sci. 2005; 18: 1519–1541.

[11] Wong GK, Liu B, Wang J, Zhang Y, Yang X, Zhang Z, et al A genetic variation map for chicken with 2.8 million single nucleotide polymorphisms. Nature. 2004; 432: 717–722. 15592405 10.1038/nature03156 PMC2263125

[12] Hermier D, Forgez P, Chapman MJ. A density gradient study of the lipoprotein and apolipoprotein distribution in the chicken, *Gallus domesticus* . Biochim Biophys Acta. 1985; 836: 105–118. 4027256 10.1016/0005-2760(85)90226-7

[13] McLeod RS, Wang Y, Wang S, Rusiño LA, Links P, Yao Z. Apolipoprotein B sequence requirements for hepatic very low density lipoprotein assembly. Evidence that hydrophobic sequences within apolipoprotein B48 mediate lipid recruitment. J Biol Chem. 1996; 271: 445–455. 10.1074/jbc.271.31.18445 8702489

[14] Zhang S, Li H, Shi H. Single marker and haplotype analysis of the chicken apolipoprotein B gene T123g and D9500D9- polymorphism reveals association with body growth and obesity. Poult Sci. 2006; 85: 178–184. 16523611 10.1093/ps/85.2.178

[15] Yamauchi T, Kamon J, Ito Y, Tsuchida A, Yokomizo T, Kita S, et al Cloning of adiponectin receptors that mediate antidiabetic metabolic effects. Nature. 2003; 423: 762–769. 12802337 10.1038/nature01705

[16] Jin Z, Pu L, Sun L, Chen W, Nan N, Li H, et al Identification of Susceptibility Variants in ADIPOR1Gene Associated with Type 2 Diabetes, Coronary Artery Disease and the Comorbidity of Type 2 Diabetes and Coronary Artery Disease. PLOS ONE. 2014; 9: e100339 10.1371/journal.pone.0100339 24967709 PMC4072681

[17] Luo N, Chung H, Garvey WT, Fu Y. Overexpression of adiponectin receptor improves the phenotypes of tallyho diabetic mice. Diabetes. 2012; 61: A415.

[18] Kershaw EE, Flier JS. Adipose Tissue as an Endocrine Organ. J Clin Endocrinol Metab. 2004; 89: 2548–2556. 15181022 10.1210/jc.2004-0395

[19] Maddineni S, Metzger S, Ocón O, Hendricks G 3rd, Ramachandran R. Adiponectin gene is expressed in multiple tissues in the chicken: food deprivation influences adiponectin messenger ribonucleic acid expression. Endocrinology. 2005; 146: 4250–4256. 15976057 10.1210/en.2005-0254

[20] Hendricks GL 3rd, Hadley JA, Krzysik-Walker SM, Prabhu KS, Vasilatos-Younken R, Ramachandran R. Unique profile of chicken adiponectin, a predominantly heavy molecular weight multimer and relationship to visceral adiposity. Endocrinology. 2009; 150: 3092–3100. 10.1210/en.2008-1558 19299452 PMC2703559

[21] Tahmoorespur M, Ghazanfari S, Nobari K. Evaluation of adiponectin gene expression in the abdominal adipose tissue of broiler chickens: feed restriction, dietary energy, and protein influences adiponectin messenger ribonucleic acid expresion. Poult Sci. 2010; 89: 2092–2100. 10.3382/ps.2010-00772 20852099

[22] Wang HB, Li H, Wang QG, Zang XY, Wang SZ, Wang YX, et al Profiling of chicken adipose tissue gene expression by genome array. BMC Genomics. 2007; 8: 193 17594506 10.1186/1471-2164-8-193 PMC1914355

[23] Barbato GF. Genetic control of food intake in chickens. J Nutr. 1994; 124: 1341S–1348S. 8064382 10.1093/jn/124.suppl_8.1341S

[24] Hood RL. The cellular basis for growth of the abdominal fat pad in broiler-type chickens. Poult Sci. 1982; 61: 117–121. 7088775 10.3382/ps.0610117

[25] Cao JJ. Effects of obesity on bone metabolism. J Orthop Surg Res. 2011; 6: 30 10.1186/1749-799X-6-30 21676245 PMC3141563

[26] Libby P, Nathan D, Abraham K, Brunzell JD, Fradkin JE, Haffner SM, et al Report of the National Heart, Lung, and Blood Institute-National Institute of Diabetes and Digestive and Kidney Diseases Working Group on Cardiovascular Complications of Type 1 Diabetes Mellitus. Circulation. 2005; 111: 3489–3493. 15983263 10.1161/CIRCULATIONAHA.104.529651

[27] Ewing B, Green P. Base-calling of automated using sequencer traces Phred. II, Error probabilities. Genome Res. 1998; 8: 186–194. 9521922

[28] Gordon D, Abajian C, Green P. Consed: A graphical tool for sequence finishing. Genome Res. 1998; 8: 195–202. 9521923 10.1101/gr.8.3.195

[29] Nickerson DA, Tobe VO, Taylor SL. PolyPhred: automating the detection and genotyping of single nucleotide substitutions using fluorescence-based resequencing. Nucleic Acids Res. 1997; 25: 2745–2751. 9207020 10.1093/nar/25.14.2745 PMC146817

[30] Vincze T, Posfai J, Roberts RJ. NEBcutter: A program to cleave DNA with restriction enzymes. Nucleic Acids Res. 2003; 31: 3688–3691. 12824395 10.1093/nar/gkg526 PMC168933

[31] SAS INSTITUTE. SAS User’s Guide: Statistics. SAS Institute Inc., Cary, NC 1464p; 2001.

[32] Gilmour AR, Gogel BJ, Cullis BR, Thompson R. ASReml User Guide Release 3.0 VSN International Ltd, Hemel Hempstead, HP1 1ES, UK 90p. 2009. Available: http://www.vsni.co.uk.

[33] Feng BZ, Wong WWL, Gao X, Schenkel F. Generalized genetic association study with samples of related individuals. Ann Appl Stat. 2011; 5: 2109–2130.

[34] Johnson RC, Nelson GW, Troyer JL, Lautenberger JA, Kessing BD, Winkler CA, et al Accounting for multiple comparisons in a genome-wide association study (GWAS). BMC Genomics. 2010; 11: 724 10.1186/1471-2164-11-724 21176216 PMC3023815

[35] Gao X, Starmer J, Martin ER. A multiple testing correction method for genetic association studies using correlated single nucleotide polymorphisms. Genet Epidemiol. 2008; 32: 361–369. 10.1002/gepi.20310 18271029

[36] Mignon-Grasteau S, Piles M, Varona L, Rochambeau H, Poivey JP, Blasco A, et al Genetic analysis of growth curve parameters for male and female chickens resulting from selection on shape of growth curve. J Anim Sci. 2000; 78: 2515–2524. 11048915 10.2527/2000.78102515x

[37] Vieira SL, Moran ET Jr. Broiler yields using chicks from egg weight extremes and diverse strains. J Appl Poult Res. 1998; 7: 339–346.

[38] Dalanezi JA, Mendes AA, Garcia EA, Garcia RG, Moreira J, Paz ICLA. Efeito da idade da matriz sobre o desempenho e rendimento de carcaça de frangos de corte. Arq Bras Med Vet Zootec. 2005; 57: 250–260.

[39] Pisto P, Santaniemi M, Turpeinen JP, Ukkola O, Kesäniemi YA. Adiponectin concentration in plasma is associated with muscle fiber size in healthy middle-aged men. Scand J Clin Lab Invest. 2012; 72: 395–402. 10.3109/00365513.2012.687759 22900744

[40] Dransfield E, Sosnicki AA. Relationship between muscle growth and poultry meat quality. Poult Sci. 1999; 78: 743–746. 10228972 10.1093/ps/78.5.743

[41] Gonnelli S, Caffarelli C, Del Santo K, Cadirni A, Guerriero C, Lucani B, et al The Relationship of Ghrelin and Adiponectin with Bone Mineral Density and Bone Turnover Markers in Elderly Men. Calcif Tissue Int. 2008; 83:55–60. 10.1007/s00223-008-9149-y 18563283

[42] Sinsigalli NA, Mcmurtry JP, Cherry JA, Siegel PB. Glucose tolerance, plasma insulin and immunore active glucagon in chickens selected for high and low body weight. The J Nutr. 1987; 117: 941–947. 3295145 10.1093/jn/117.5.941

[43] Gonzales E, Buyse J, Takita TS, Sartori JR, Decuypere E. Metabolic Disturbances in Male Broilers of Different Strains. 1. Performance, Mortality, and Right Ventricular Hypertrophy. Poult Sci. 1998; 77: 1646–1653. 9835338 10.1093/ps/77.11.1646

[44] Ninov K, Ledur MC, Alves HJ, Rosário MF, Nones K, Coutinho LH. Investigation of leptin gene in broiler and layer chicken lines. Sci Agric. 2008; 65: 214–219.

[45] Crespo N, Esteve-Garcia E. Dietary Fatty Acid Profile Modifies Abdominal Fat Deposition in Broiler Chickens. Poult Sci. 2001; 80: 71–78. 11214339 10.1093/ps/80.1.71

